# [Pyr^1^]Apelin-13_(1–12)_ Is a Biologically Active ACE2 Metabolite of the Endogenous Cardiovascular Peptide [Pyr^1^]Apelin-13

**DOI:** 10.3389/fnins.2017.00092

**Published:** 2017-02-28

**Authors:** Peiran Yang, Rhoda E. Kuc, Aimée L. Brame, Alex Dyson, Mervyn Singer, Robert C. Glen, Joseph Cheriyan, Ian B. Wilkinson, Anthony P. Davenport, Janet J. Maguire

**Affiliations:** ^1^Department of Medicine, Experimental Medicine and Immunotherapeutics, University of CambridgeCambridge, UK; ^2^Division of Medicine, Bloomsbury Institute of Intensive Care Medicine, University College LondonLondon, UK; ^3^Department of Chemistry, Centre for Molecular Informatics, University of CambridgeCambridge, UK; ^4^Department of Surgery and Cancer, Biomolecular Medicine, Imperial College LondonLondon, UK

**Keywords:** apelin, apelin receptor, [Pyr1]apelin-13(1–12), ACE2, human heart, pulmonary arterial hypertension, forearm plethysmography, biased signaling

## Abstract

**Aims:** Apelin is a predicted substrate for ACE2, a novel therapeutic target. Our aim was to demonstrate the endogenous presence of the putative ACE2 product [Pyr^1^]apelin-13_(1–12)_ in human cardiovascular tissues and to confirm it retains significant biological activity for the apelin receptor *in vitro* and *in vivo*. The minimum active apelin fragment was also investigated.

**Methods and Results:** [Pyr^1^]apelin-13 incubated with recombinant human ACE2 resulted in de novo generation of [Pyr^1^]apelin-13_(1–12)_ identified by mass spectrometry. Endogenous [Pyr^1^]apelin-13_(1–12)_ was detected by immunostaining in human heart and lung localized to the endothelium. Expression was undetectable in lung from patients with pulmonary arterial hypertension. In human heart [Pyr^1^]apelin-13_(1–12)_ (pK_i_ = 8.04 ± 0.06) and apelin-13(F13A) (pK_i_ = 8.07 ± 0.24) competed with [^125^I]apelin-13 binding with nanomolar affinity, 4-fold lower than for [Pyr^1^]apelin-13 (pK_i_ = 8.83 ± 0.06) whereas apelin-17 exhibited highest affinity (pK_i_ = 9.63 ± 0.17). The rank order of potency of peptides to inhibit forskolin-stimulated cAMP was apelin-17 (pD_2_ = 10.31 ± 0.28) > [Pyr^1^]apelin-13 (pD_2_ = 9.67 ± 0.04) ≥ apelin-13(F13A) (pD_2_ = 9.54 ± 0.05) > [Pyr^1^]apelin-13_(1–12)_ (pD_2_ = 9.30 ± 0.06). The truncated peptide apelin-13(R10M) retained nanomolar potency (pD_2_ = 8.70 ± 0.04) but shorter fragments exhibited low micromolar potency. In a β-arrestin recruitment assay the rank order of potency was apelin-17 (pD_2_ = 10.26 ± 0.09) >> [Pyr^1^]apelin-13 (pD_2_ = 8.43 ± 0.08) > apelin-13(R10M) (pD_2_ = 8.26 ± 0.17) > apelin-13(F13A) (pD_2_ = 7.98 ± 0.04) ≥ [Pyr^1^]apelin-13_(1–12)_ (pD_2_ = 7.84 ± 0.06) >> shorter fragments (pD_2_ < 6). [Pyr^1^]apelin-13_(1–12)_ and apelin-13(F13A) contracted human saphenous vein with similar sub-nanomolar potencies and [Pyr^1^]apelin-13_(1–12)_ was a potent inotrope in paced mouse right ventricle and human atria. [Pyr^1^]apelin-13_(1–12)_ elicited a dose-dependent decrease in blood pressure in anesthetized rat and dose-dependent increase in forearm blood flow in human volunteers.

**Conclusions:** We provide evidence that ACE2 cleaves [Pyr^1^]apelin-13 to [Pyr^1^]apelin-13_(1–12)_ and this cleavage product is expressed in human cardiovascular tissues. We have demonstrated biological activity of [Pyr^1^]apelin-13_(1–12)_ at the human and rodent apelin receptor *in vitro* and *in vivo*. Our data show that reported enhanced ACE2 activity in cardiovascular disease should not significantly compromise the beneficial effects of apelin based therapies for example in PAH.

## Introduction

Apelins are a family of peptides that activate the apelin receptor (also known as APJ) and have an emerging importance in the physiology and pathophysiology of the cardiovascular system (Yang et al., 2015). Apelin peptides are present in human vascular and cardiac endothelial cells (Kleinz and Davenport, 2004) and plasma, with [Pyr^1^]apelin-13 identified as the most abundant cardiovascular isoform (De Mota et al., 2004; Maguire et al., 2009; Zhen et al., 2013). Apelins mediate three major actions *in vitro*. Interaction with the apelin receptor on cardiac myocytes causes increased cardiac contractility and inotropic action, with apelin an order of magnitude more potent than endothelin-1. In vessels with an intact endothelium, apelin acts to release vasodilators that may oppose the actions of vasoconstrictors. We have also shown that removal of endothelium unmasks a constrictor response mediated by apelin receptors present on the vascular smooth muscle (Maguire et al., 2009). Importantly, in healthy volunteers and heart failure patients, the major effect of apelin infused into the forearm *in vivo* was nitric oxide dependent arterial dilatation (Japp et al., 2008, 2010; Barnes et al., 2013; Brame et al., 2015). In heart failure patients, intracoronary [Pyr^1^]apelin-13 caused coronary vasodilatation and increased cardiac contractility (Japp et al., 2010; Barnes et al., 2013). Systemic infusions of [Pyr^1^]apelin-13 in both volunteers and patients increased cardiac index and lowered mean arterial blood pressure and peripheral vascular resistance (Japp et al., 2010; Barnes et al., 2013). Apelin is down-regulated in pulmonary arterial hypertension (PAH), a devastating disease characterized by vascular remodeling resulting in progressive obliteration of the pulmonary circulation, leading to right ventricle (RV) hypertrophy and right heart failure (Alastalo et al., 2011; Chandra et al., 2011). Therefore the apelin receptor may represent a novel target for future drug development.

Human angiotensin converting enzyme 2 (ACE2) has 40% sequence similarity with the C-terminal dipeptidyl-peptidase, ACE (Donoghue et al., 2000; Tipnis et al., 2000). ACE2 is expressed for example in heart, kidney and lung (Donoghue et al., 2000; Hamming et al., 2004) and is implicated in pathological conditions such as heart failure where it is up-regulated (Zisman et al., 2003; Goulter et al., 2004). A major role of ACE2 is to degrade angiotensin II to angiotensin (1–7) which then acts as a beneficial vasodilator and anti-proliferation agent, counter-balancing the actions of the vasoconstrictor angiotensin II (Santos et al., 2003, 2008). ACE2 is also a viral receptor for the severe acute respiratory syndrome coronavirus (Li et al., 2008) which down-regulates the enzyme from the cell surface resulting in angiotensin II-induced lung injury (Kuba et al., 2005). This has been the rational for the development of recombinant human ACE2 (rhACE2) in clinical trials for acute lung injury (Haschke et al., 2013). Interestingly, enhancing ACE2 activity pharmacologically or by gene transfer was effective in preventing or reversing PAH (Shenoy et al., 2011; Dai et al., 2015). Some beneficial actions of ACE2 are thought to be mediated by the conversion of angiotensin II to angiotensin(1–7). However, the possibility of interaction of ACE2 with other peptides was not clear until a screen of over 120 biologically active peptides reported only two others to be hydrolyzed with high catalytic efficiency by ACE2; dynorphin A 1-13, which has no reported vasoactivity and apelin-13, or apelin-36, resulting in the removal of the C-terminal phenylalanine, producing the metabolites apelin-13_(1–12)_ or apelin-36_(1–35)_ (Vickers et al., 2002). The loss of the terminal phenylalanine in apelin has been assumed to be a mechanism of degradation and inactivation of the peptide. Our aim was to understand the impact of this ACE2 cleavage reaction on the apelin signaling pathway.

**Figure 1:**
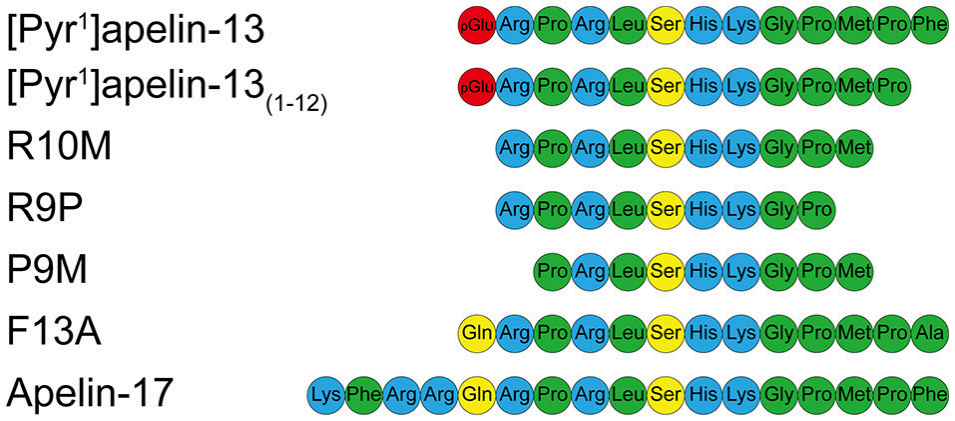
**Aligned amino acid sequences of apelin peptides**. Hydrophobic amino acids are shown in green, uncharged polar amino acids in yellow, basic amino acids in blue and pyroglutamate in red.

Specifically, our objectives were firstly to confirm that [Pyr^1^]apelin-13_(1–12)_ (Figure 1) can be produced by ACE2 hydrolysis of [Pyr^1^]apelin-13 and find evidence that [Pyr^1^]apelin-13_(1–12)_ is an endogenous peptide and determine its distribution in human cardiovascular tissues. Secondly, to demonstrate that [Pyr^1^]apelin-13_(1–12)_ binds to the apelin receptor and can activate down-stream signaling pathways in cell based assays. Thirdly, we have determined that [Pyr^1^]apelin-13_(1–12)_ retains significant biological activity, compared with our previously reported data (Maguire et al., 2009; Brame et al., 2015) for [Pyr^1^]apelin-13, *in vitro* using vascular and cardiac human and rodent tissues and by systemic infusions of [Pyr^1^]apelin-13_(1–12)_ *in vivo* in the rat; finally, since the predominant action of apelin infused into the human forearm is vasodilatation, we have performed first-in-man studies with [Pyr^1^]apelin-13_(1–12)_ to explore the physiological action of the peptide in healthy volunteers. We also wished to explore the structure activity relationship (SAR) of the apelin peptides and therefore additional experiments were performed with the N-terminal extended putative endogenous peptide apelin-17, alanine substituted apelin-13(F13A), and the shorter fragments apelin-13(R10M), apelin-13(R9P) and apelin-13(P9M) (Figure 1). Our data expand our knowledge on the structure activity relationship of apelin peptides and demonstrate the significant biological activity of the ACE2 metabolite [Pyr^1^]apelin-13_(1–12)_. These data support the hypothesis that therapeutic strategies enhancing ACE2 activity or up-regulation of ACE2 in cardiovascular disease, both of which may result in enhanced breakdown of [Pyr^1^]apelin-13, may not significantly compromise the beneficial effects of endogenous apelin signaling via generation of [Pyr^1^]apelin-13_(1–12)_.

## Methods

### Cardiovascular tissue collection

Human tissues samples were obtained with informed consent (Papworth Hospital Research Tissue Bank REC08/H0304/56) and local ethical approval (REC05/Q0104/142).

### Synthesis of [Pyr^1^]apelin-13_(1–12)_ from [Pyr^1^]apelin-13

Synthesis of [Pyr^1^]apelin-13_(1–12)_ from [Pyr^1^]apelin-13 was confirmed by incubating [Pyr^1^]apelin-13 (5 nmol) with rhACE2 enzyme (5 pmol, GSK, Ware, UK) in buffer (pH 6.5) containing 2-(N-morpholino)ethanesulfonic acid (MES, 500 mmol/L), NaCl (300 mmol/L) and ZnCl_2_ (10 μmol/L) for 2 h at 37°C followed by quenching with 10 μmol ethylenediaminetetraacetic acid (EDTA) (see Vickers et al., 2002 for similar protocol). The reaction mixture was analyzed by Maldi-TOF mass spectrometry after samples were desalted using Millipore μC18ZipTip (MA, USA), washed with 5% acetic acid and eluted with CHCA matrix (in 50% aqueous acetonitrile containing 0.1% trifluoroacetic acid) to a stainless steel sample slide. Samples were air dried and analyzed using a Waters Maldi MicroMX time-of-flight mass spectrometer (MA, USA). Calibration was external using polyethylene glycol. The 10 Hz-laser power was just above threshold. The spectra were the sum of 1,000 shots collected from a spiral track of the sample area. Data were processed using Waters MassLynx software (MA, USA). Control reactions included incubation of [Pyr^1^]apelin-13_(1–12)_ (5 nmol) with rhACE2, incubation of [Pyr^1^]apelin-13 (5 nmol) or [Pyr^1^]apelin-13_(1–12)_ (5 nmol) without rhACE2 and incubation of rhACE2 (5 pmol) without peptides.

### Localization of endogenous [Pyr^1^]apelin-13_(1–12)_ by immunostaining

**Figure 2:**
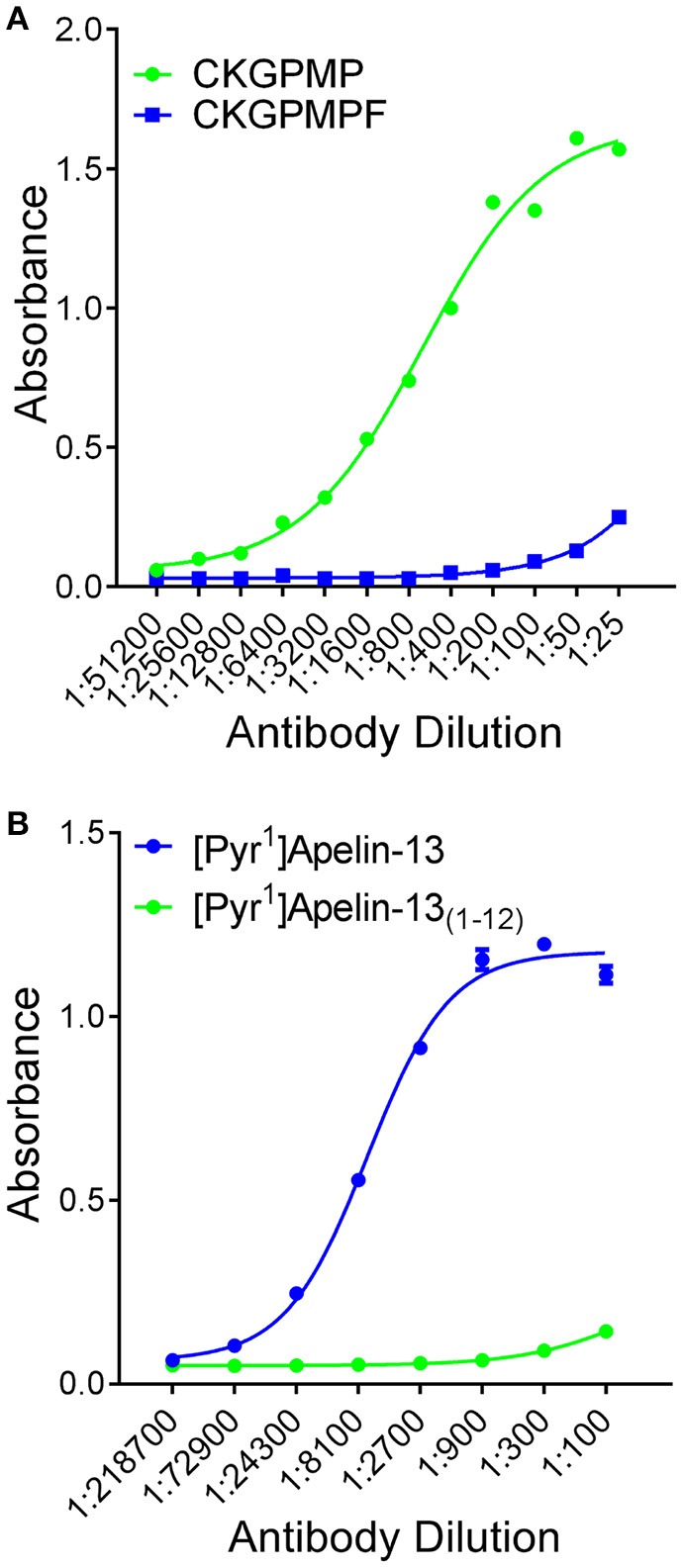
**Selectivity of antisera determined by ELISA. (A)** [Pyr^1^]apelin-13_(1–12)_ antiserum demonstrated selectivity to the antigen CKGPMP (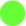, corresponding to [Pyr^1^]Apelin-13_(1–12)_) relative to CKGPMPF (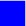, corresponding to [Pyr^1^]Apelin-13). **(B)** The apelin-12 antiserum demonstrated selectivity to [Pyr^1^]Apelin-13 (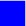) relative to [Pyr^1^]Apelin-13_(1–12)_ (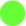).

Peroxidase-anti-peroxidase and dual-labeling immunofluorescent staining were conducted as described (Kleinz et al., 2005) using frozen sections of human cardiomyopathy heart (*n* = 4), histologically normal (*n* = 6) and PAH (*n* = 4) lung. Affinity purified rabbit-anti-[Pyr^1^]apelin-13_(1–12)_ antiserum (1:25–1:100 dilution) was custom synthesized (antibody raised against the relevant apelin peptide fragment CKGPMP, that lacks the C-terminal phenylalanine) and selectivity confirmed by comparison with the corresponding fragment containing the C-terminal phenylalanine (CKGPMPF) by ELISA (Figure 2A). In contrast the commercially available apelin-12 antibody (Phoenix Pharmaceuticals Inc. CA, USA) crossed reacted with [Pyr^1^]apelin-13 but not [Pyr^1^]apelin-13_(1–12)_ (Figure 2B). ACE2 (1:50, R&D Systems, MN, USA and 1:200, Abcam, Cambridge, UK) antisera were also used. Von Willebrand factor (vWF) (1:50, Dako, Glostrup, Denmark) was used as an endothelial marker. The peroxidase stained sections were examined with a bright field microscope (Olympus, Southend-on-Sea, UK) and imaged using a CC12 camera and CellD Soft Imaging System (Olympus), whereas fluorescent staining were imaged using a Leica Scanning Confocal Microscope (TCS SP2, Leica Microsystems, Milton Keynes, UK). Image processing with rolling ball method and histogram redistribution were applied equally to the entire image and channel overlay were carried out using ImageJ.

### Competition binding assays

Assays were performed in human heart as previously described (Brame et al., 2015). Briefly, homogenate of human left ventricle (LV) was incubated for 90 min with 0.1 nmol/L [Glp^65^, Nle^75^, Tyr^77^][^125^I]apelin-13 in assay buffer (mmol/L; Tris 50, MgCl_2_ 5, pH 7.4, 22°C), in the presence of increasing concentrations of [Pyr^1^]apelin-13, [Pyr^1^]apelin-13_(1–12)_, apelin-13(F13A), and apelin-17 (0.01 nmol/L–100 μmol/L) or, for SAR, a single concentration (1 μmol/L) apelin-13(R10M), apelin-13(R9P) and apelin-13(P9M). Non-specific binding was defined using 2 μmol/L [Pyr^1^]apelin-13. Equilibrium was broken by centrifugation (20,000 g for 10 min, 4°C) and pellets washed with Tris-HCl buffer (50 mmol/L, pH 7.4, 4°C), re-centrifuged and pellets counted for detection of bound radioactivity. Competition binding data were analyzed using GraphPad Prism 6 (GraphPad Software, Inc. La Jolla, CA, USA) to obtain values of pK_i_ (the negative log_10_ of the dissociation constant derived from the IC_50_ for the competing ligands, the radioligand concentration and radioligand affinity by the Cheng and Prusoff equation). Experiments were performed in triplicate.

### Cell-based functional assays

Inhibition of cAMP accumulation, β-arrestin recruitment and receptor internalization by apelin isoforms, modified and truncated apelin peptides were studied using cells expressing the human apelin receptor (DiscoverX, CA, USA) as per the manufacturer's instructions. In all assays the resulting chemiluminescent signal was measured as relative light units (RLU) using a LumiLITE™ Microplate Reader (DiscoveRx, Fremont, CA). In the cAMP assay 15 μmol/L forskolin was used to stimulate cAMP production and concentration-response curves were constructed to [Pyr^1^]apelin-13, [Pyr^1^]apelin-13_(1–12)_, apelin-17, apelin-13(F13A) (all 1 pmol/L–30 nmol/L), apelin-13(R10M), apelin-13 (R9P) and apelin-13(P9M) (all 1 nmol/L–10 μmol/L). Agonist responses were expressed as a % of the forskolin response. In the β-arrestin and internalization assays concentration-response curves were constructed to [Pyr^1^]apelin-13, [Pyr^1^]apelin-13_(1–12)_, apelin-13(F13A), apelin-13(R10M), (all 10 pmol/L–1 μmol/L), apelin-17 (1 pmol/L-100 nmol/L), apelin-13 (R9P) and apelin-13(P9M) (both 1 nmol/L–300 μmol/L). Agonist responses were expressed as a % of the maximum response to [Pyr^1^]apelin-13. Data were analyzed using a 4-parameter logistic equation using GraphPad Prism 6 to determine values of potency, pD_2_ (−log_10_ EC_50_, where EC_50_ is the concentration producing half maximal response) and maximum response (E_MAX_). n-Values are given as number of replicates/number of experiments.

Using the data from the cAMP and β-arrestin assays with the predominant cardiac isoform [Pyr^1^]apelin-13 used as the reference ligand, the relative activation of G protein-dependent and -independent signaling pathways by [Pyr^1^]apelin-13, [Pyr^1^]apelin-13_(1–12)_ and apelin-17 were compared using bias analysis as described by van der Westhuizen et al. (2014).

Additional β-arrestin assay experiments were performed with [Pyr^1^]apelin-13 that had been incubated with ACE2 as described above. In this assay control concentration-response curves were constructed to [Pyr^1^]apelin-13 and [Pyr^1^]apelin-13_(1–12)_ and these were compared to concentration-response curves constructed to both agonists following pre-incubation with rhACE2.

### *In vitro* functional studies

Vascular smooth muscle apelin receptor-mediated contraction was exploited in a bioassay to compare the *in vitro* potency of apelin peptides. Experiments were carried out as previously described (Maguire, 2002) in endothelium-denuded saphenous vein with concentration-response curves constructed to [Pyr^1^]apelin-13_(1–12)_ and apelin-13(F13A) (1 pmol/L–300 nmol/L). Agonist responses were expressed as a % of a terminal response to KCl (100 mmol/L). The inotropic action of [Pyr^1^]apelin-13_(1–12)_ was determined in mouse paced RV (*n* = 6) and for comparison in two samples of human paced atrial appendage strips as described (Maguire et al., 2009). Data were expressed as % of the terminal response to CaCl_2_. Data from vascular and cardiac experiments were analyzed using a 4-prameter logistic curve (GraphPad Prism 6) to determine values of pD_2_ and E_MAX_.

### Systemic infusions in rat and echocardiography

All experiments were performed according to local ethics committee (University College, London) and Home Office (UK) guidelines under the 1986 Scientific Procedures Act and conformed to the Directive 2010/63/EU. The effects of systemic infusion of [Pyr^1^]apelin-13_(1–12)_ (incremental bolus doses 1–300 nmol/300 μL) on blood pressure, heart rate, stroke volume and cardiac output were assessed in male Wistar rats (300 ± 25 g body weight) as described (Brame et al., 2015), that were anesthetized with isoflurane (5% induction, 2% maintenance, continuous monitoring throughout). The left carotid artery and right jugular vein were cannulated (0.96 mm polyvinyl chloride tubing). Mean arterial pressure (MAP) was measured throughout the procedure via a pressure transducer (Powerlab AD Instruments, Chalgrove, UK) connected to the arterial line. Baseline hemodynamics were recorded using Chart 7.0 acquisition software and a 16 channel Powerlab system (AD Instruments, Chalgrove, UK) after a 30 min stabilization period. Thoracic echocardiography was performed at a scanning depth of 0–2 cm using a 14 MHz probe (Vivid 7 Dimension, GE Healthcare, Bedford, UK). Pulsed-wave Doppler was used to determine aortic blood flow velocities in the aortic arch. Stroke volume (SV) was determined as the product of the velocity–time integral (VTI) and vessel cross-sectional area. Data from six consecutive cardiac cycles were used to determine heart rate (HR) and a marker of left ventricular contractility, peak velocity (PV). Values of SV and HR were used to calculate cardiac output (CO). Respiration rate was determined from movement of the diaphragm using time-motion (M)-model. At the end of the study rats were euthanized by intravenous pentobarbitone and exsanguination.

### Forearm venous occlusion plethysmography in human volunteers

Studies were performed in healthy volunteers (*n* = 12) in the University of Cambridge Vascular Research Unit, Addenbrooke's Hospital, Cambridge, UK. Volunteer characteristics are given in Table 1. This study was carried out in accordance with the recommendations of the National Research Ethics Service Committee East of England-Cambridge Central with written informed consent from all subjects. All subjects gave written informed consent in accordance with the Declaration of Helsinki. The protocol was approved by the National Research Ethics Service Committee East of England-Cambridge Central (REC 11/EE/0305). Changes in forearm blood flow (FBF) in response to [Pyr^1^]apelin-13_(1–12)_ (1, 10, 100 nmol/min) were measured as previously described (Brame et al., 2015).

**Table 1:** **Volunteer characteristics**.

***N* =**	**12**
Age (y)	27 ± 2
Gender (m/f)	6/6
Height (cm)	173 ± 3
Weight (kg)	70.4 ± 2.8
BMI (kg/m^2)^	23.6 ± 1
HR (bpm)	68 ± 3
SBP (mmHg)	127 ± 6
DBP (mmHg)	72 ± 4
MAP (mmHg)	90 ± 4

*Values are mean ± SEM*.

Exclusion criteria were ischemic heart disease, respiratory, renal or neurological disease, diabetes mellitus, hypertension, BMI > 30, BMI <18; smoker; pregnant; use of vasoactive medication or NSAIDS/aspirin within 48 h of study; current involvement in other research studies. An Omron HEM705CP oscillimetric sphygmomanometer was used to measure blood pressure and heart rate at baseline and then every 6 min in the contralateral arm. Periodically, a cuff around the upper arm was inflated for ~8 s to 40 mmHg then deflated for 4 s to interrupt venous return and during a 3 min measurement hand circulation was excluded by inflation of wrist cuffs to 200 mmHg. Changes in forearm volume were measured by mercury-in-silastic strain gauge with FBF subsequently expressed as ml/100 ml forearm volume per min. For infusion of peptides via a 16-gauge catheter (Portex, Kent, UK), the brachial artery (non-dominant arm) was cannulated (27-gauge needle, Cooper's Needle Works, Birmingham, UK) under local anesthesia (lignocaine 1%, Hameln Pharmaceuticals Ltd., Gloucester, UK). FBF was measured in both arms and the response to [Pyr^1^]apelin-13_(1–12)_ presented as absolute change in forearm blood flow from the pre-infusion baseline value.

[Pyr^1^]apelin-13_(1–12)_, supplied in sealed glass vials and stored at −40°C until required, was allowed to warm to room temperature and diluted with physiological saline to produce stock solutions that were then filtered (0.2 μm flat filter, Portex, Hythe, UK) before further dilution in saline. [Pyr^1^]apelin-13_(1–12)_ was infused in three incremental doses during each visit. Doses were previously optimized in a pilot study. Each dose was infused for 6 min with a 20 min saline infusion washout period before the next dose was administered. At the end of the study sodium nitroprusside was infused at 3μg/min for 6 min as a positive control followed by a saline infusion as a negative control.

### Statistical analyses

Measurements are mean ± standard error of the mean (SEM). Data analysis and statistical testing were performed using GraphPad Prism 6 to determine values of affinity (pK_i_) calculated from competition IC_50_ values using the Cheng and Prusoff equation, potency [pD_2_ (−log_10_ EC_50_, the concentration producing 50% of maximum response)] and maximum response (E_MAX_) as appropriate. Cell assay pD_2_ values were compared by one-way ANOVA followed by Tukey's multiple comparison test. For rat *in vivo* experiments the effect of [Pyr^1^]apelin-13_(1–12)_ on BP, PV and VTI were expressed as % change from vehicle control in the same animal with other variables (SV and CO) expressed in absolute values. The effect of increasing doses of [Pyr^1^]apelin-13_(1–12)_ on each parameter was compared to baseline vehicle control using repeated measures one way ANOVA followed by Dunnett's multiple comparison test. Similarly in the human FBF study the response to successive increasing doses of [Pyr^1^]apelin-13_(1–12)_ was compared to pre-infusion baseline value using repeated measures one-way ANOVA followed by Dunnett's multiple comparison test.

### Materials

All chemical reagents were purchased from Sigma (Poole, UK), unless otherwise stated. [Pyr^1^]apelin-13_(1–12)_ was custom synthesized to GLP standard using Fmoc chemistry on a solid phase support matrix to 98% purity by Maldi-TOF Mass spectroscopy and RP-HPLC analysis. Peptides were tested for sterility and demonstrated to be pyrogen free and biological activity confirmed using the β-arrestin assay. All apelin peptides were synthesized by Severn Biotech (Kidderminster, UK).

## Results

### [Pyr^1^]apelin-13_(1–12)_ is synthesized from [Pyr^1^]apelin-13 by ACE2

**Figure 3:**
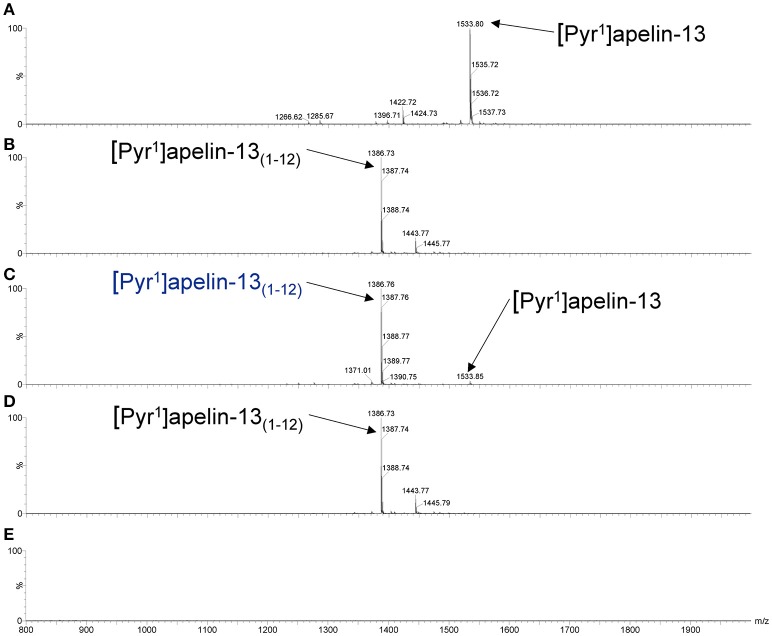
**Cleavage of [Pyr^1^]apelin-13 to [Pyr^1^]apelin-13_(1–12)_ by rhACE2 *in vitro***. Maldi-TOF spectra for **(A)** [Pyr^1^]apelin-13, **(B)** [Pyr^1^]apelin-13_(1–12)_, **(C)** [Pyr^1^]apelin-13 incubated with rhACE2, **(D)** [Pyr^1^]apelin-13_(1–12)_ incubated with rhACE2, **(E)** rhACE2 alone.

As shown in the mass spectra, [Pyr^1^]apelin-13 (Figure 3A) and [Pyr^1^]apelin-13_(1–12)_ (Figure 3B) alone produced signals at 1533.8 and 1386.7 m/z, respectively. Incubating [Pyr^1^]apelin-13 with rhACE2 resulted in a signal at the mass-to-charge ratio of 1386.7, corresponding to de novo generation of [Pyr^1^]apelin-13_(1–12)_, with a weak signal representing the remaining parent peptide (Figure 3C). In contrast, incubating [Pyr^1^]apelin-13_(1–12)_ with rhACE2 (Figure 3D) did not produce shorter apelin fragments. Finally, rhACE2 enzyme alone (Figure 3E) did not result in interfering signals in the relevant mass range.

### [Pyr^1^]apelin-13_(1–12)_ is an endogenous apelin peptide localized to the endothelium

**Figure 4:**
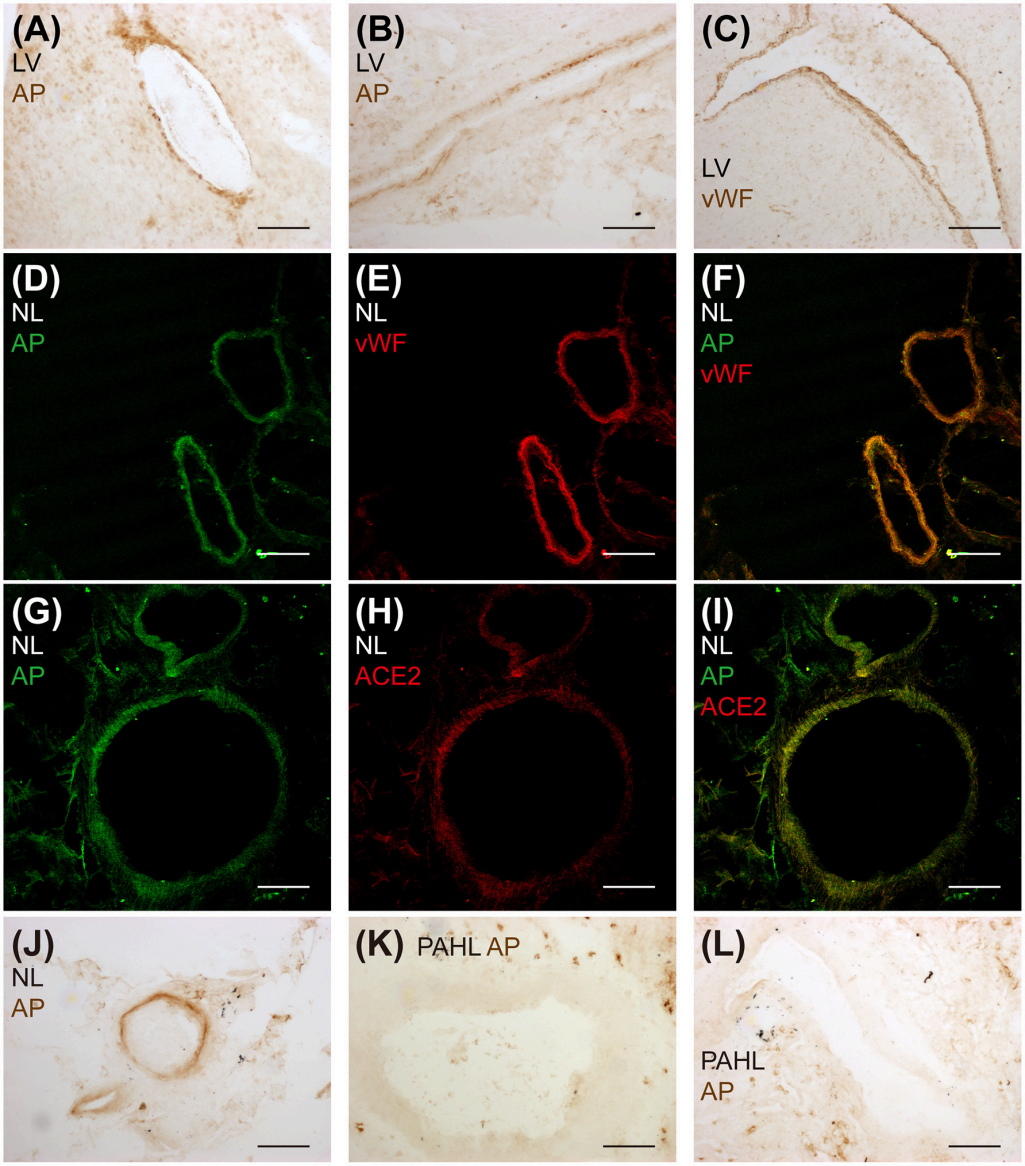
**Detection of endogenous [Pyr^1^]apelin-13_(1–12)_ peptide in human cardiovascular tissues**. Bright field microphotographs of [Pyr^1^]apelin-13_(1–12)_-LI in **(A)** vascular and **(B)** endocardial endothelium and **(C)** vWF-LI in cardiomyopathy human heart. Confocal microphotographs of **(D)** [Pyr^1^]apelin-13_(1–12)_-LI (green), **(E)** vWF-LI (red) and **(F)** their overlay in normal human lung. **(G)** [Pyr^1^]apelin-13_(1–12)_-LI (green), **(H)** ACE2-LI (red) and **(I)** their overlay in normal human lung. Bright field microphotographs of **(J)** [Pyr^1^]apelin-13_(1–12)_-LI in normal human lung and **(K,L)** the absence of [Pyr^1^]apelin-13_(1–12)_-LI in PAH human lung tissue. LV, cardiomyopathy human heart; AP, [Pyr^1^]apelin-13_(1–12)_; NL, normal human lung; PAHL, PAH human lung. Scale bar = 200 μm.

Endogenous [Pyr^1^]apelin-13_(1–12)_ peptide was detectable in human cardiovascular tissues and localized to the endothelium (Figure 4). [Pyr^1^]apelin-13_(1–12)_-like immunoreactivity (-LI) was detected in vascular (Figure 4A) and endocardial (Figure 4B) endothelium identified by positive staining with vWF (Figure 4C) in sections of human cardiomyopathy heart, where ACE2 expression has been reported to be increased (Zisman et al., 2003; Goulter et al., 2004). [Pyr^1^]apelin-13_(1–12)_-LI (Figure 4D) and vWF-LI (Figure 4E) were also co-expressed (Figure 4F) in human lung. Importantly, [Pyr^1^]apelin-13_(1–12)_-LI (Figure 4G) and ACE2-LI (Figure 4H) co-localized (Figure 4I) in pulmonary blood vessels. Importantly, as apelin is reduced in PAH, the presence of [Pyr^1^]apelin-13_(1–12)_ was investigated in sections of human PAH lung. Compared to normal lung (Figure 4J), [Pyr^1^]apelin-13_(1–12)_-LI was not detectable in the vascular endothelium of PAH lung (Figures 4K,L).

### [Pyr^1^]apelin-13_(1–12)_ binds to and activates the human apelin receptor

**Figure 5:**
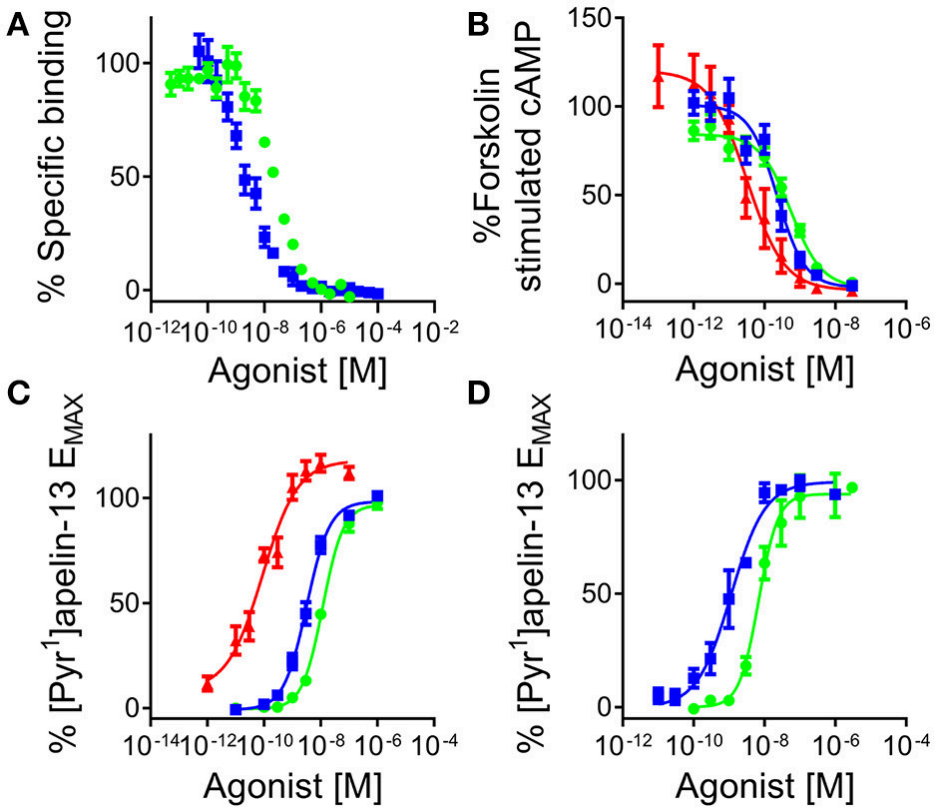
**[Pyr^1^]apelin-13_(1–12)_ binds to and activates the human apelin receptor. (A)** Competition binding curve for [Pyr^1^]apelin-13 (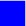) and [Pyr^1^]apelin-13_(1–12)_ (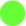) in human left ventricle (*n* = 3). In cell based assays [Pyr^1^]apelin-13 (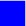), [Pyr^1^]apelin-13_(1–12)_ (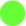), and apelin-17 (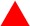) **(B)** inhibited forskolin-stimulated cAMP production; **(C)** induced β-arrestin recruitment and **(D)** triggered apelin receptor internalization.

[Pyr^1^]apelin-13_(1–12)_ competed with [^125^I]apelin-13 binding with nanomolar affinity, pK_i_ = 8.04 ± 0.06 (*n* = 3), that was 4-fold lower than the parent molecule [Pyr^1^]apelin-13 (pK_i_ = 8.83 ± 0.06, *n* = 3) (Figure 5A). Apelin-13(F13A) exhibited comparable affinity to [Pyr^1^]apelin-13_(1–12)_ (pK_i_ = 8.07 ± 0.24, *n* = 3) whereas the extended peptide apelin-17 competed with highest affinity (pK_i_ = 9.63 ± 0.17, *n* = 3). Of the shorter fragments apelin-13(R10M) (1 μM) competed for 100% of specific binding whereas apelin-13(R9P) and apelin-13(P9M) were less effective competing for 38% and 62% respectively.

In the signaling assays, [Pyr^1^]apelin-13_(1–12)_ inhibited forskolin-stimulated cAMP production with sub-nanomolar potency, pD_2_ = 9.30 ± 0.06 (*n* = 8/3) and was 2-fold less potent than the reference agonist [Pyr^1^]apelin-13, pD_2_ = 9.67 ± 0.04 (*n* = 7/5) but comparable to apelin-13(F13A) (pD_2_ = 9.54 ± 0.05, *n* = 2/1). Apelin-17 (pD_2_ = 10.31 ± 0.28, *n* = 5/2) was ~5 times more potent than [Pyr^1^]apelin-13. All ligands fully inhibited cAMP production (Figure 5B). Apelin-13(R10M) retained nanomolar potency (pD_2_ = 8.70 ± 0.04, *n* = 3/1) whereas inhibition was incomplete for apelin-13(R9P) and apelin-13(P9M) at 10 μM.

In the G protein-independent β-arrestin recruitment assay [Pyr^1^]apelin-13 (pD_2_ = 8.43 ± 0.08, *n* = 25/10), [Pyr^1^]apelin-13_(1–12)_ (pD_2_ = 7.84 ± 0.06, *n* = 17/6) and apelin-13(F13A) (pD_2_ = 7.98 ± 0.04, *n* = 6/2) were 15-40-fold less potent than in the cAMP assay, with [Pyr^1^]apelin-13 ~4-fold more potent than [Pyr^1^]apelin-13_(1–12)_ and apelin-13(F13A). Interestingly, unlike the shorter peptides, apelin-17 exhibited comparable potency as an agonist in both the β-arrestin and cAMP assays with a pD_2_ = 10.26 ± 0.09 (*n* = 11/4) (Figure 5C). Apelin-13(R10M) (pD_2_ = 8.26 ± 0.17, *n* = 9/3) was ~2-fold less potent than [Pyr^1^]apelin-13 and curves were incomplete for apelin-13(R9P) and aplin-13(P9M) at 300 μM.

Similar to the β-arrestin assay, in the internalization assay [Pyr^1^]apelin-13_(1–12)_ (pD_2_ = 8.19 ± 0.06) was 5-fold less potent than [Pyr^1^]apelin-13 (pD_2_ = 8.94 ± 0.17). Both peptides were full agonists with comparable efficacy values (E_MAX_ values were 97 ± 2% and 99 ± 3% respectively) (Figure 5D).

Comparing the cAMP and β-arrestin data for the three endogenous peptides with [Pyr^1^]apelin-13 as the reference agonist, analysis (Table 2) demonstrated a bias factor of 0.24 for [Pyr^1^]apelin-13_(1–12)_ and 68 for apelin-17 indicating that compared to [Pyr^1^]apelin-13, [Pyr^1^]apelin-13_(1–12)_ was 4-fold G protein biased and apelin-17 was markedly β-arrestin biased.

**Table 2:** **Pathway bias analysis for [Pyr^1^]apelin-13_(1–12)_ and apelin-17 compared to [Pyr^1^]apelin-13**.

**Pathway**		**[Pyr^1^]apelin-13**	**[Pyr^1^]apelin-13_(1–12)_**	**Apelin-17**
cAMP	LogR	9.70 ± 0.05	9.43 ± 0.10	10.28 ± 0.23
	ΔLogR	0.00 ± 0.08	−0.20 ± 0.11	0.48 ± 0.24
	RE	1	0.63	3
β-Arrestin	LogR	8.02 ± 0.13	7.89 ± 0.17	10.34 ± 0.19
	ΔLogR	0.00 ± 0.06	−0.82 ± 0.10	2.32 ± 0.07
	RE	1	0.15	208
cAMP vs. β-Arrestin	ΔΔLogR	0.00 ± 0.10	−0.62 ± 0.15	1.83 ± 0.25
	Bias Factor	1	0.24	68

In the β-arrestin recruitment assay [Pyr^1^]apelin-13 was significantly more potent than [Pyr^1^]apelin-13_(1–12)_ (*p* < 0.0001), [Pyr^1^]apelin-13 incubated with rhACE2 (*p* < 0.001) or [Pyr^1^]apelin-13_(1–12)_ incubated with rhACE2 (*p* < 0.0001). Both of the rhACE2 combinations exhibited comparable potency to [Pyr^1^]apelin-13_(1–12)_ (*p* > 0.05) (Table 3). Therefore the reaction product of [Pyr^1^]apelin-13 and rhACE2 more closely resembled [Pyr^1^]apelin-13_(1–12)_ than [Pyr^1^]apelin-13.

**Table 3:** **Relative potencies of apelin peptides without and with pre-incubation with recombinant human ACE2 in a β-arrestin assay**.

	**Potency (pD_2_)**	**E_MAX_ (% [Pyr^1^]apelin-13)**
[Pyr^1^]apelin-13	8.82 ± 0.03^†^	103 ± 4
[Pyr^1^]apelin-13+rhACE2	7.81 ± 0.10^***^	80 ± 7
[Pyr^1^]apelin-13_(1–12)_	7.27 ± 0.30^****^	98 ± 5
[Pyr^1^]apelin-13_(1–12)_+rhACE2	7.42 ± 0.04^****^	97 ± 3

Significantly different from [Pyr^1^]apelin-13;

*** *P < 0.001*,

**** *P < 0.0001*.

Significantly different from [Pyr^1^]apelin-13_(1–12)_;

† *P < 0.001. One-way ANOVA with Tukey's multiple comparisons test*.

### Cardiovascular actions of apelin peptides *In vitro*

**Figure 6:**
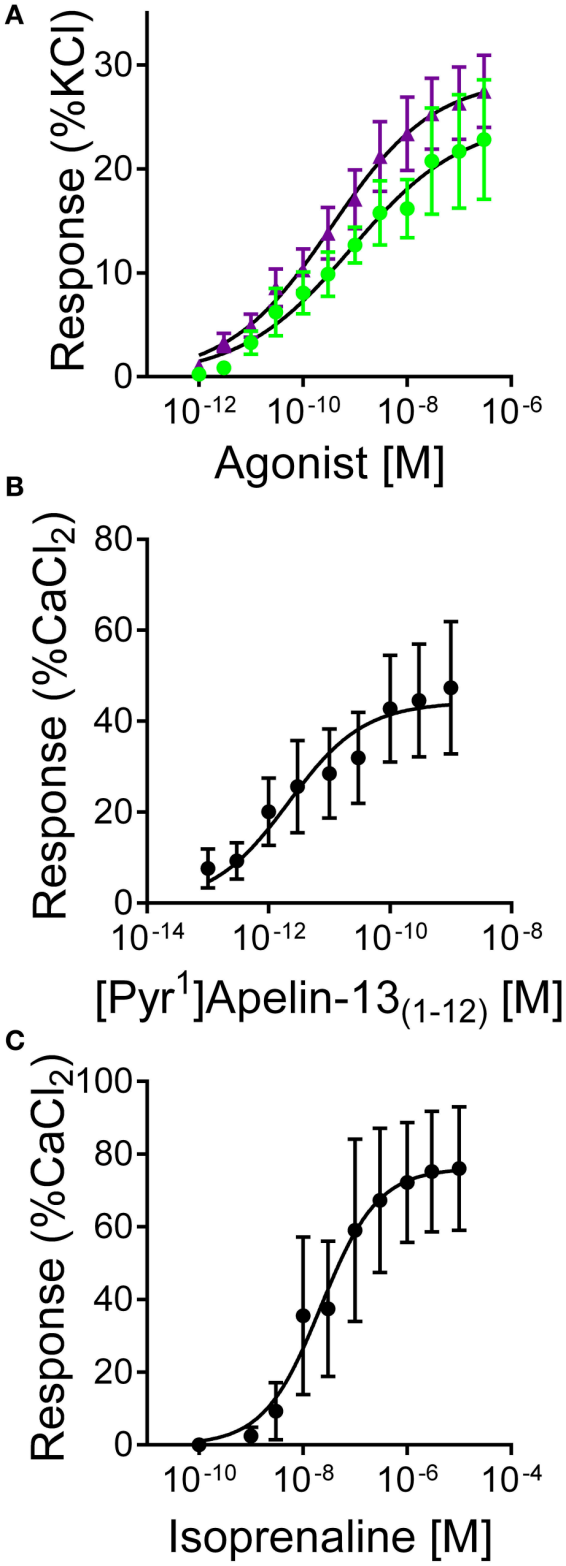
**Responses to (A)** [Pyr^1^]apelin-13_(1–12)_ (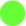, *n* = 9) and apelin-13(F13A) (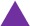, *n* = 9) in endothelium-denuded human saphenous vein *in vitro*. **(B)** Inotropic responses to **(B)** [Pyr^1^]apelin-13_(1–12)_ (*n* = 6) and **(C)** isoprenaline (*n* = 4) in mouse paced right ventricle.

[Pyr^1^]apelin-13_(1–12)_ (pD_2_ = 9.63 ± 0.35, E_MAX_ = 24 ± 6%, *n* = 9) and apelin-13(F13A) (pD_2_ = 9.72 ± 0.36, E_MAX_ = 23 ± 4%, *n* = 9) contracted saphenous vein with comparable sub-nanomolar potencies and maximum responses (Figure 6A) to the data that we had previously obtained with [Pyr^1^]apelin-13 in this assay (Brame et al., 2015).

In mouse paced RV [Pyr^1^]apelin-13_(1–12)_ produced a concentration-dependent increase in force of contraction with pD_2_ = 11.68 ± 0.33, E_MAX_ 49 ± 16% CaCl_2_ (*n* = 6) (Figure 6B), compared to isoprenaline with pD_2_ = 7.88 ± 0.42, E_MAX_ = 78 ± 17% CaCl_2_ (*n* = 4) (Figure 6C). Limited human atrial appendage strips were available to test for the inotropic action of [Pyr^1^]apelin-13_(1–12)_. In tissue from two patients [Pyr^1^]apelin-13_(1–12)_ acted as a positive inotrope with a sub-nanomolar potency value (pD_2_ = 9.61, E_MAX_ = 38% CaCl_2_, *n* = 2).

### Cardiovascular actions of apelin peptides *In vivo*

**Figure 7:**
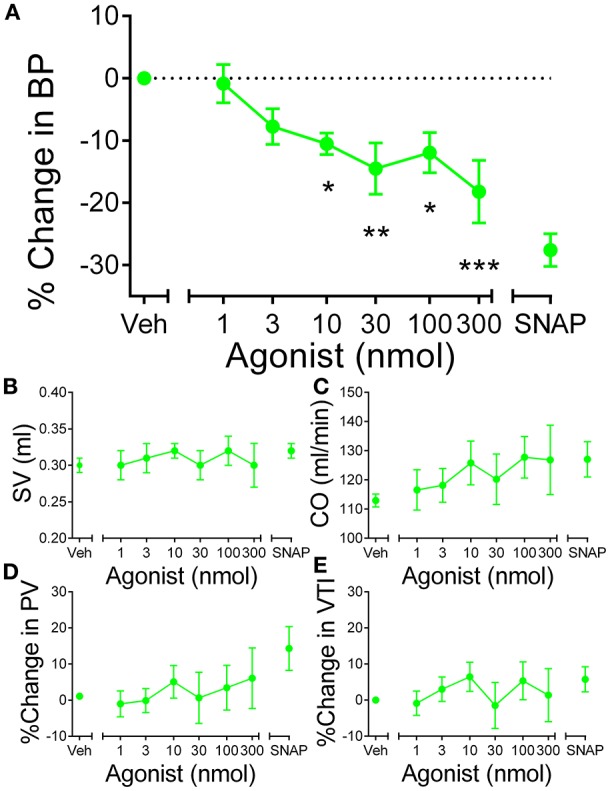
***In* vivo actions of [Pyr^1^]apelin-13_(1–12)_ in rat. (A)** Administration of intravenous [Pyr^1^]apelin-13_(1–12)_ (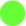, *n* = 5) resulted in a significant dose-dependent decrease in blood pressure in rat *in vivo*. Other parameters **(B)** stroke volume (SV), **(C)** cardiac output (CO), **(D)** peak velocity (PV) and velocity-time interval (VTI) were unaffected at any dose of [Pyr^1^]apelin-13_(1–12)._ Significantly different from baseline; ^*^P < 0.05, ^**^P < 0.01, ^***^P < 0.001. One-way ANOVA repeated measures compared to baseline with Dunnett's multiple comparison test.

In anesthetized rat, [Pyr^1^]apelin-13_(1–12)_ (*n* = 5) elicited a dose-dependent decrease in BP (Figure 7A). The decrease in BP was significantly different (*P* < 0.0001) from baseline at doses of 10–300 nmol. Other cardiac parameters, SV, CO, PV and VTI were not altered by [Pyr^1^]apelin-13_(1–12)_ (Figures 7B–E).

**Figure 8:**
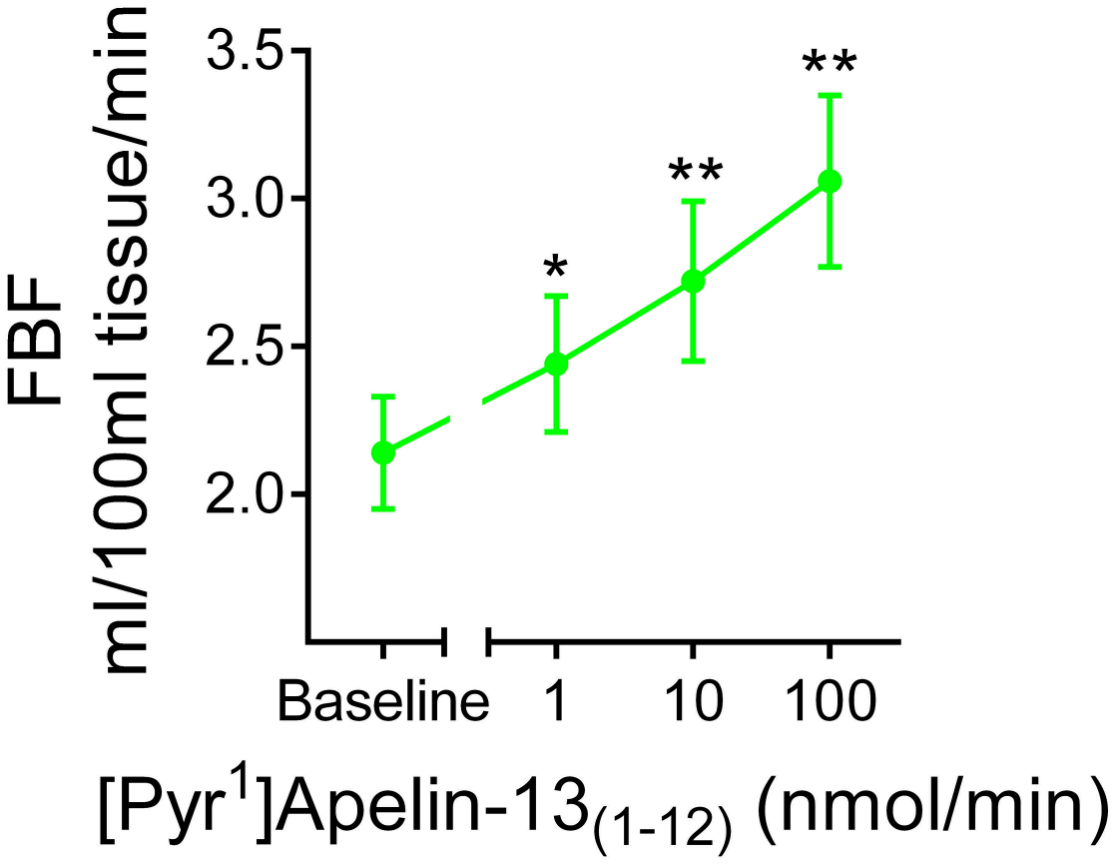
***In vivo* actions of [Pyr^1^]apelin-13_(1–12)_ in human volunteers**. Infusion of [Pyr^1^]apelin-13_(1–12)_ in healthy human volunteers (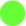, *n* = 12) resulted in a dose-dependent increase in forearm blood flow. Significantly different from baseline; ^*^P < 0.05, ^**^P < 0.01. One-way ANOVA repeated measures compared to baseline with Dunnett's multiple comparison test.

In human volunteers (*n* = 12), [Pyr^1^]apelin-13_(1–12)_ (Figure 8) produced comparable dose-dependent increases in forearm blood flow to that which we have shown for [Pyr^1^]apelin-13 (Brame et al., 2015). No significant effects of either peptide on heart rate or blood pressure were observed at any dose (data not shown).

## Discussion

We have characterized the synthesis, receptor pharmacology and functional activity of the ACE2 metabolite of [Pyr^1^]apelin-13, [Pyr^1^]apelin-13_(1–12)_, *in vitro* and *in vivo*. For the first time we have detected endogenous [Pyr^1^]apelin-13_(1–12)_ in human cardiovascular tissues and demonstrated biological activity of this metabolite *in vitro* and in a first-in-man study.

### Synthesis of [Pyr^1^]apelin-13_(1–12)_ from [Pyr^1^]apelin-13 by ACE2

Apelin-13 and apelin-36 peptides were previously reported to be substrates of purified human ACE2 enzyme (Vickers et al., 2002). In this study, we confirmed (Wang et al., 2016) that ACE2 also catalyzed the conversion of [Pyr^1^]apelin-13, the predominant cardiovascular form of apelin, to [Pyr^1^]apelin-13_(1–12)_ *in vitro*. This result was not unexpected since the PMPF sequence of [Pyr^1^]apelin-13 (Figure 1) conforms to the consensus sequence for ACE2-mediated hydrolysis; Pro-X_(1–3*residues*)_-Pro-Hydrophobic (Vickers et al., 2002). In support, in the β-arrestin assay the product of *de novo* ACE2 metabolism demonstrated comparable potency to synthetic [Pyr^1^]apelin-13_(1−12)_(Table 3). Our results are consistent with a study reporting that a small proportion of [Pyr^1^]apelin-13 was cleaved into [Pyr^1^]apelin-13_(1–12)_ in rat *in vivo* (Murza et al., 2014).

We have previously demonstrated endothelial expression of apelin (Kleinz and Davenport, 2004; Kleinz et al., 2005) using an antibody specific for apelin isoforms containing the C-terminal phenylalanine (Figure 2B). This is consistently reported by others using alternative strategies to detect apelin expression (Sheikh et al., 2008). ACE2 is also expressed in the endothelium (Donoghue et al., 2000; Hamming et al., 2004), raising the possibility of endothelial processing of [Pyr^1^]apelin-13 to produce [Pyr^1^]apelin-13_(1–12)_. For this study, we generated an antibody that cross-reacts with apelin isoforms without the C-terminal phenylalanine that has allowed selective identification of [Pyr^1^]apelin-13_(1–12)_ in human heart and lung. [Pyr^1^]apelin-13_(1–12)_-LI was specifically localized to the endothelium, where the apelin receptor was also expressed, suggesting the possibility of autocrine signaling by this metabolite in these tissues. Importantly, in human PAH lung, where endothelial apelin expression is known to be reduced (Alastalo et al., 2011; Kim et al., 2013), no staining for [Pyr^1^]apelin-13_(1–12)_ was detected.

### Biological activity of [Pyr^1^]apelin-13_(1–12)_

ACE2-mediated hydrolysis has been assumed to inactivate apelin peptides. Wang and colleagues have recently reported that the ACE2 product of [Pyr^1^]apelin-13 exhibits reduced or absent cardiovascular actions compared to the parent molecule (Wang et al., 2016). However, emerging evidence from structure activity studies suggested that the C-terminal phenylalanine was not a critical residue for apelin biological activity (Fan et al., 2003; Medhurst et al., 2003) and our hypothesis was that compared to [Pyr^1^]apelin-13, [Pyr^1^]apelin-13_(1–12)_ may retain significant activity. Indeed, our results showed that [Pyr^1^]apelin-13_(1–12)_ has nanomolar affinity for the native human apelin receptor exhibiting only a 3-fold reduction in binding affinity compared the parent peptide. This is consistent with previous studies where the C-terminal phenylalanine of [Pyr^1^]apelin-13 or apelin-13 was replaced with alanine (F13A) (Fan et al., 2003; Medhurst et al., 2003) or removed from apelin-17 (K16P)(El Messari et al., 2004; Iturrioz et al., 2010) with only minimal loss of receptor affinity. Similarly, substitution of the C-terminal phenylalanine of [Pyr^1^]apelin-13 with D-phenylalanine resulted in only a 20-fold decrease in receptor binding affinity, which was modest compared to substitution of other residues known to be important for binding (Murza et al., 2012). Overall, these studies show that loss of the C-terminal phenylalanine from apelin isoforms does not significantly alter receptor binding.

The apelin receptor is G_αi_-coupled (Habata et al., 1999) therefore we next showed that [Pyr^1^]apelin-13_(1–12)_ inhibited forskolin-stimulated cAMP with potency only 2-fold less than [Pyr^1^]apelin-13. This is in agreement with the previous reports of alanine and D-phenylalanine substituted [Pyr^1^]apelin-13 (Medhurst et al., 2003; Murza et al., 2012) and K16P (El Messari et al., 2004; Iturrioz et al., 2010; Ceraudo et al., 2014). Therefore, our study confirms that G_αi_-induced signaling is preserved in response to [Pyr^1^]apelin-13_(1–12)_ receptor binding. In addition to G protein-dependent signaling, activation of the apelin receptor causes β-arrestin recruitment and receptor internalization (Evans et al., 2001). In these assays, [Pyr^1^]apelin-13_(1–12)_ again behaved as a full agonist at the human apelin receptor with only a 5-fold reduction in potency compared to [Pyr^1^]apelin-13. This ability to induce receptor internalization without the C-terminal phenylalanine was reported for apelin-13(F13A) (Fan et al., 2003), however, K16P was shown by a second group to exhibit markedly reduced potency and efficacy in β-arrestin recruitment and internalization of rat apelin receptor (El Messari et al., 2004; Iturrioz et al., 2010; Ceraudo et al., 2014). Importantly, compared to [Pyr^1^]apelin-13, [Pyr^1^]apelin-13_(1–12)_ is not a biased agonist in G protein-dependent and -independent signaling.

Apelins are modulators of vascular tone and cardiac contractility *in* vitro and *in vivo* (Japp et al., 2008, 2010; Maguire et al., 2009; Barnes et al., 2013; Brame et al., 2015). Therefore, as expected we found that [Pyr^1^]apelin-13_(1–12)_ contracted human saphenous vein with equal potency and efficacy compared with our previous data for [Pyr^1^]apelin-13 (Maguire et al., 2009; Brame et al., 2015) and was a potent inotrope in mouse paced right ventricle and human paced atria. We next investigated the *in vivo* actions of [Pyr^1^]apelin-13_(1–12)_. In anesthetized rat compared to our previously published data for [Pyr^1^]apelin-13 (Brame et al., 2015), the ACE2 metabolite caused a smaller but significant drop in blood pressure which contrasts with Wang and colleagues (Wang et al., 2016) who reported that [Pyr^1^]apelin-13, but not [Pyr^1^]apelin-13_(1–12)_, showed a sustained effect on blood pressure for up to 60 min after administration to mice *in vivo*, although both peptides appear to show an initial comparable reduction in blood pressure over the first 10–15 min in these experiments. Interestingly, these authors also showed that [Pyr^1^]apelin-13 is rapidly cleaved by ACE2 *in vitro* and *in vivo* suggesting that the ACE2 cleavage product may contribute to their observed sustained response to [Pyr^1^]apelin-13 and as might be expected the half-life of the cleavage product is likely to be short and therefore the response to infused [Pyr^1^]apelin-13_(1–12)_ is observed for only the initial 10~15 min of the experiment. In agreement with this hypothesis, in our rat studies (Brame et al., 2015) the half-life of [Pyr^1^]apelin-13 was less than 3 min and in humans less than 8 min (Japp et al., 2008). Importantly, in a first-in-man study, infusion of [Pyr^1^]apelin-13_(1–12)_ resulted in an increase in forearm blood flow that was the same magnitude as we had previously obtained with [Pyr^1^]apelin-13 (Brame et al., 2015), confirming that this metabolite is a vasodilator in humans *in vivo*.

### Structure activity relationships of apelin peptides

The 55-amino acid proapelin, derived from the 77-residue prepropeptide, contains a number of paired basic amino acids residues that are possible cleavage sites for endopeptidases to produce 13 to 36-residue isoforms (Tatemoto et al., 1998; Habata et al., 1999). While the identities of these peptidases remain unknown, a study reported the direct cleavage of proapelin to apelin-13 by proprotein convertase subtilisin/kexin 3 (PCSK3, or furin), bypassing the generation of longer isoforms (Shin et al., 2013). Post-translational modification results in the predominant apelin isoform detected in human cardiovascular tissue and plasma, [Pyr^1^]apelin-13 (De Mota et al., 2004; Kleinz and Davenport, 2004; Maguire et al., 2009; Zhen et al., 2013). The putative ACE2 metabolite of this cardiac peptide, [Pyr^1^]apelin-13_(1–12)_, is the focus of our study but we have also explored the pharmacology of several other important apelin isoforms to better understand the apelin structure activity relationships, namely the minimum active fragment apelin-13(R10M), apelin-13(F13A) and apelin-17.

As described above, amino acid substitution studies have proposed a consensus on those amino acids within apelin-13 that are important for receptor binding and activation (Narayanan et al., 2015). The C-terminal phenylalanine (F13), adjacent proline and the N-terminal pyroglutamic acid were not identified as important. We have investigated whether the truncated peptides apelin-13(R10M), apelin-13(R9P) and apelin-13(P9M) retained significant binding and functional activity with the hypothesis that apelin-13(R10M) was likely to represent the minimum active fragment as it was the shortest fragment containing all the identified important amino acids. Results of the competition binding experiments and β-arrestin recruitment assays supported this hypothesis as do data from previous publications showing diminished G_αi_-mediated signaling and calcium mobilization when the methionine (position 11 in apelin-13) was removed (Medhurst et al., 2003; Zhang et al., 2014), while others have demonstrated activity of apelin-12 (apelin-13 without the N-terminal pyroglutamic acid) *in vivo* (Pisarenko et al., 2011). Residues arginine 2 and methionine 11 are indispensable as they are required to form the crucial RPRL motif or provide steric volume (Langelaan et al., 2009; Macaluso and Glen, 2010; Gerbier et al., 2015). In contrast, although the C-terminal phenylalanine has been shown to make specific contacts within the binding pocket of the apelin receptor (Iturrioz et al., 2010), our experimental data suggested that its removal did not abolish binding or functional activity.

Early alanine scanning studies indicated that replacing the C-terminal phenylalanine with alanine did not abolish G_αi_-mediated signaling, calcium mobilization (Medhurst et al., 2003) and receptor internalization (Fan et al., 2003). However, conflicting data have been reported for F13A, suggesting that it was an antagonist of the apelin receptor in terms of G_αi_-mediated signaling ([Pyr^1^]apelin-13(F13A); De Mota et al., 2000) and hypotensive effect (apelin-13(F13A); Lee et al., 2005). In this study, we found that apelin-13(F13A) resembled [Pyr^1^]apelin-13_(1–12)_ in receptor binding, cell based assays and the vasoconstrictor bioassay. Based on our results apelin-13(F13A) behaves as an apelin agonist with no evidence of receptor antagonism.

Apelin-17, despite its unclear biosynthetic pathway, has been reported to have equal or higher binding affinity and potency in inhibiting cAMP accumulation and inducing receptor internalization compared with [Pyr^1^]apelin-13 (Medhurst et al., 2003; El Messari et al., 2004). We investigated apelin-17 in our assays and consistently found higher binding affinity and higher potency than [Pyr^1^]apelin-13 in the cAMP and β-arrestin assays. Intriguingly, apelin-17 appeared to be more biased for β-arrestin compared to G protein signaling relative to the reference agonist [Pyr^1^]apelin-13. This suggests that N-terminal extension of apelin-13 may result in peptides that stabilize different conformations of the apelin receptor and may be a mechanism by which apelin receptor activation is fine-tuned at the cellular level.

### Implication on the interactions between the apelin and renin-angiotensin systems

**Figure 9:**
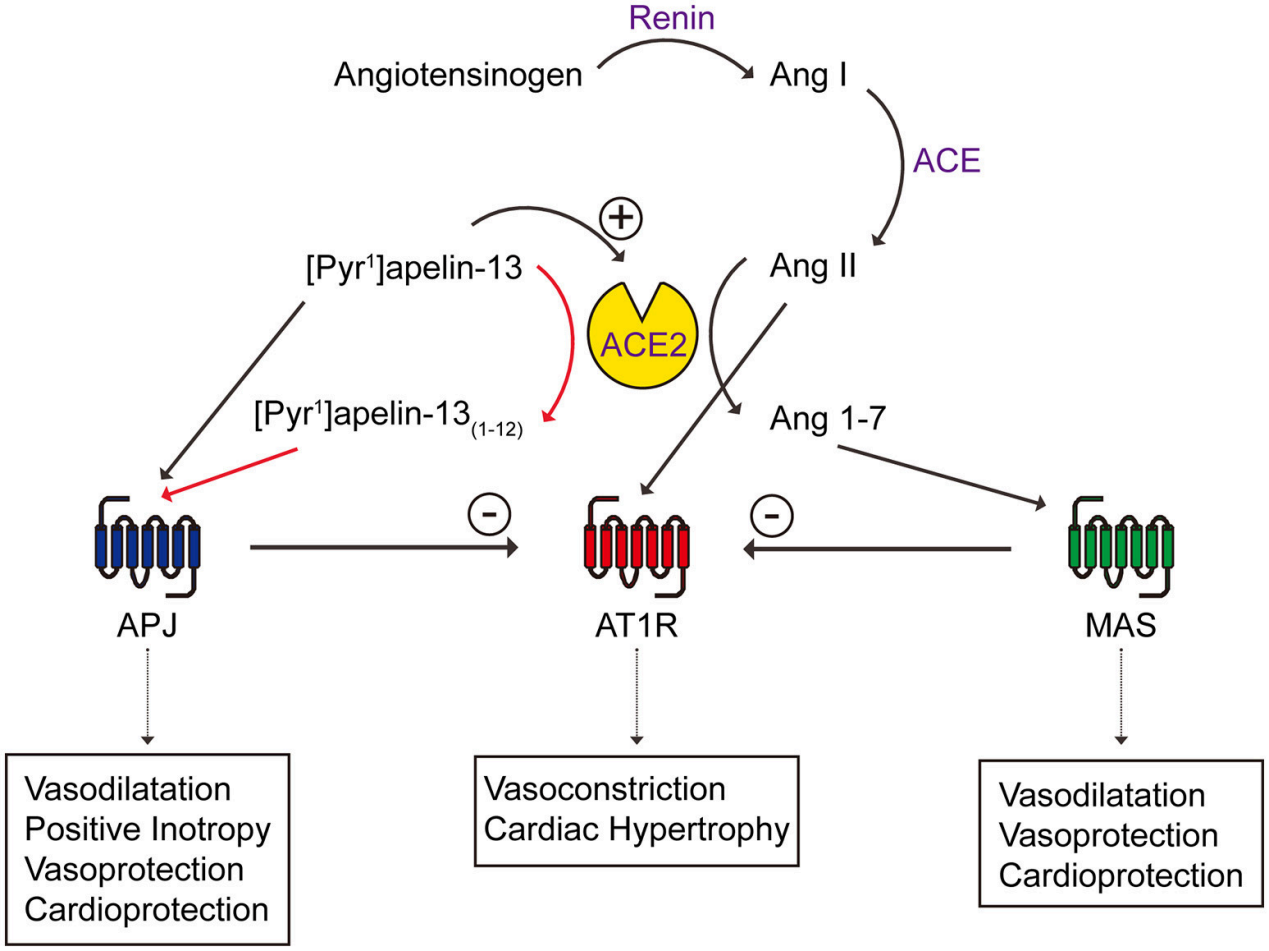
**Schematic diagram showing the interactions between apelin/apelin receptor with ACE2 of the renin angiotensin system**. New contributions of this study are shown in red arrows. Ang, angiotensin; APJ, apelin receptor; AT1R, angiotensin receptor type I; MAS, Mas receptor.

Past studies have suggested a possible link between apelin, its receptor, ACE2 and the renin angiotensin system. For example, apelin knockout mice showed aging or stress-associated cardiac contractility defects, similar to the cardiac phenotype of ACE2 knockout mice (Kuba et al., 2007) and apelin receptor knockout mice are more sensitive to the pressor effect of angiotensin II (Ishida et al., 2004). Interestingly, ACE2 expression is reduced in apelin knockout mice and apelin up-regulates ACE2 expression, indicating that apelin may reciprocally impact ACE2 (Sato et al., 2013). At the receptor level, the apelin receptor has been shown to physically interact with the angiotensin receptor type I (AT1R) (Chun et al., 2008), forcing it into a low affinity state and reducing the binding and signaling of angiotensin II (Siddiquee et al., 2013). Reduced apelin expression due to heart failure or angiotensin II administration can be restored by AT1R blockade (Iwanaga et al., 2006). The opposing effects of apelin/ACE2 and angiotensin II have been shown in health and diseases such as heart failure, atherosclerosis and obesity/diabetes (Ashley et al., 2006; Gurzu et al., 2006; Iwanaga et al., 2006; Zhong et al., 2007; Chun et al., 2008; Barnes et al., 2013; Siddiquee et al., 2013) (Figure 9). Our study contributes further evidence that ACE2, that is up-regulated in disease, acting on [Pyr^1^]apelin-13 may result in the generation of the active metabolite, [Pyr^1^]apelin-13_(1–12)_, and therefore a compensatory maintenance of apelin receptor signaling. However, a limitation of our study is that we have not measured levels of apelin peptides in plasma of patients receiving rhACE2 therapy, but we would hypothesize that levels of [Pyr^1^]apelin-13_(1–12)_ would be elevated and [Pyr^1^]apelin-13 decreased compared to controls.

In conclusion, this study confirmed that ACE2 cleaves [Pyr^1^]apelin-13 into [Pyr^1^]apelin-13_(1–12)_ and has demonstrated biological activity of [Pyr^1^]apelin-13_(1–12)_ at the human apelin receptor *in vitro* and in the cardiovascular system of rat and human *in vivo*. The results also clarify R10M as the shortest active apelin fragment, apelin-13(F13A) as an agonist and apelin-17 as a more potent agonist than [Pyr^1^]apelin-13. Therefore, our study shows that reported enhanced ACE2 activity in cardiovascular disease should not significantly compromise the beneficial effects of apelin based therapies for example in PAH.

## Author contributions

All authors contributed either to the conception or design of the work (PY, AB, AD, MS, RG, JC, IW, APD, and JM), or acquired and analyzed data or interpreted data (PY, RK, AB, AD, MS, APD, and JM). All authors agree to be accountable for all aspects of the work.

## Funding

This work was supported by the Wellcome Trust Programme in Metabolic and Cardiovascular Disease [096822/Z/11/Z to APD]; Wellcome Trust [WT 107715 to APD]; Medical Research Council [MC_PC_14116 to APD]; Wellcome Trust Programme in Translational Medicines and Therapeutics [085686 to APD], and in part by the National Institute for Health Research Cambridge Biomedical Research Centre; and the Pulmonary Hypertension Association UK.

### Conflict of interest statement

The authors declare that the research was conducted in the absence of any commercial or financial relationships that could be construed as a potential conflict of interest.
